# Electrosprayed Stearic-Acid-Coated Ethylcellulose Microparticles for an Improved Sustained Release of Anticancer Drug

**DOI:** 10.3390/gels9090700

**Published:** 2023-08-29

**Authors:** Yuexin Ji, Hua Zhao, Hui Liu, Ping Zhao, Deng-Guang Yu

**Affiliations:** 1 School of Materials and Chemistry, University of Shanghai for Science and Technology, No. 516 Jungong Road, Shanghai 200093, China; 213353172@st.usst.edu.cn (Y.J.); huiliu@usst.edu.cn (H.L.); 203613027@st.usst.edu.cn (P.Z.); 2 Medical School, Quzhou College of Technology, No. 18 Jiangyuan Road, Quzhou 324000, China

**Keywords:** sustained release, anticancer drug, ethylcellulose, coaxial electrospraying, stearic acid, microparticle, anticancer, insoluble gels

## Abstract

Sustained release is highly desired for “efficacious, safe and convenient” drug delivery, particularly for those anticancer drug molecules with toxicity. In this study, a modified coaxial electrospraying process was developed to coat a hydrophobic lipid, i.e., stearic acid (SA), on composites composed of the anticancer drug tamoxifen citrate (TC) and insoluble polymeric matrix ethylcellulose (EC). Compared with the electrosprayed TC-EC composite microparticles M1, the electrosprayed SA-coated hybrid microparticles M2 were able to provide an improved TC sustained-release profile. The 30% and 90% loaded drug sustained-release time periods were extended to 3.21 h and 19.43 h for M2, respectively, which were significantly longer than those provided by M1 (0.88 h and 9.98 h, respectively). The morphology, inner structure, physical state, and compatibility of the components of the particles M1 and M2 were disclosed through SEM, TEM, XRD, and FTIR. Based on the analyses, the drug sustained-release mechanism of multiple factors co-acting for microparticles M2 is suggested, which include the reasonable selections and organizations of lipid and polymeric excipient, the blank SA shell drug loading, the regularly round shape, and also the high density. The reported protocols pioneered a brand-new manner for developing sustained drug delivery hybrids through a combination of insoluble cellulose gels and lipid using modified coaxial electrospraying.

## 1. Introduction

Most of the anticancer drugs, regardless of the active biomolecules (such as curcumin, quercetin, silybum marianum, and paclitaxel) or the synthetic therapeutic molecules, on the one hand, are poorly water soluble or even insoluble [1,2,3,4]. On the other hand, they are toxic due to a high blood drug concentration after oral administration resulting from the initial burst release, which is particularly a negative case for numerous nano/micro drug delivery systems [5,6,7,8]. Thus, better drug dissolution and sustained release of these drugs after oral delivery is highly desired for a “safe, efficacious, and convenient” delivery to the patients [9,10,11,12].

For the sustained release of a drug, the common strategies that can be relied upon can be divided into two approaches. One is to encapsulate the drug molecules into an insoluble inert matrix [13,14]. Some examples are phospholipid, insoluble polymers, biodegradable polymers, and also many inorganic materials, such as silicon, carbon nano tubes, and graphene [15,16,17]. The other is to treat the drug and the excipients through advanced pharmaceutical techniques, which are frequently introduced into the field of pharmaceuticals from other material conversion methods [18,19,20,21]. Certainly, these two approaches are frequently integrated together to develop a wide variety of drug sustained-release DDSs.

Cellulose and its derivatives are highly popular as polymeric matrices for providing all types of drug controlled-release profiles in many fields, such as drug delivery, tissue engineering, and food packaging engineering [22,23,24,25,26,27]. In general, soluble cellulose derivatives can be exploited for promoting the fast release of the poorly water-soluble drugs, such as a series of hydroxypropyl methylcellulose [28]. Insoluble cellulose derivatives are often exploited for drug extended- or sustained-release profiles, such as ethylcellulose (EC) and cellulose acetate (CA) [29,30]. These cellulose derivatives have fine film-forming, filament-forming, and other processing properties [31]. Thus, in the literature, they are frequently converted with a guest drug to form new kinds of DDSs. Particularly in this nano era, they are often transferred into nanoparticles, nanofibers, and beads-on-a-string products through a certain pharmaceutical nanotechnology [32,33,34].

One most recent pharmaceutical nanotechnology is electrospinning, which belongs to an electrohydrodynamic atomization (EHDA) process [35,36,37,38,39]. In comparison, electrospraying, as a sister EHDA method of electrospinning, receives less attention for drug delivery applications [40,41]. In the past several years, single-fluid electrospinning has been quickly moving to coaxial, tri-axial, side-by-side, and tri-layer side-by-side processes [42,43,44,45,46,47]. Furthermore, many fluids without electrospinnability have also taken part in the multiple-fluid electrospinning processes because only one of the fluids must be electrospinnable [48]. However, the electrospraying process is still mainly the single-fluid process and also the traditional coaxial process. Inspired by modified coaxial electrospinning, in which unspinnable fluids can be explored as the sheath working fluids for creating core–sheath nanofibers [49], unsolidable fluids may also be utilized as the shell working fluids for generating core–shell particles.

Tamoxifen citrate (TC) is a non-steroidal anti-estrogen drug with a structure similar to estrogen. It mainly competes with estrogen for estrogen receptors so as to prevent estrogen from entering tumor cells, prevent estrogen from playing its role, and thus inhibit the proliferation of breast cancer cells. Its most important role is to treat recurrent and metastatic breast cancer in women and to serve as an adjuvant treatment for postoperative metastasis of breast cancer to prevent recurrence [50,51]. In addition, its anti-estrogen effect can also be used to improve breast hyperplasia, pain, and discomfort symptoms. However, there are a series of possible common adverse reactions after the oral administration of TC, which are included as follows: (1) Gastrointestinal reactions, mainly manifested as nausea, vomiting, abdominal pain, and diarrhea; (2) The main side effects of the reproductive system are menstrual disorder, menopause, and vaginal bleeding; (3) Skin side effects, manifested as facial flushing and rash; (4) Symptoms of the mental nerves, mainly manifested as headaches and dizziness; (5) Changes in blood routine can lead to a decrease in white blood cells and platelets in patients; and (6) Some patients may also experience abnormal liver function. Thus, sustained release of TC after oral administration is highly desired for the patient’s compliance and a better therapeutic effect [5,6,50,51].

In this study, we hypothesized that unsolidable, i.e., dilute stearic acid (SA) solution, can be utilized as a shell liquid to conduct a modified coaxial electrospraying process, in which the core fluid was a solidable drug polymer co-dissolved solution composed of EC and TC. We further hypothesized that the coating SA layer, as a hydrophobic shell, was able to remarkably modify the sustained-release profile of TC from the insoluble EC matrix. A series of characterizations were carried out to disclose the products’ morphologies and inner structures, the components’ physical states and their compatibility, and the targeted sustained release performances.

## 2. Results and Discussion

### 2.1. The Coaxial Electrospraying and the Core–Shell Structures in Drug Delivery

**Figure 1:**
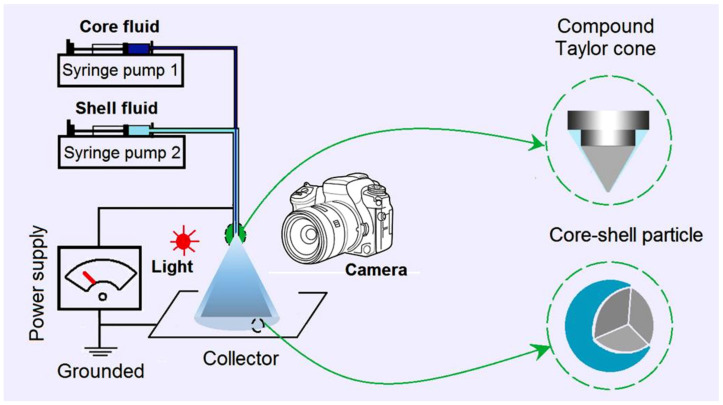
A diagram showing the modified coaxial electrospraying system, its main components, and key information about the working process.

Just as coaxial electrospinning is used for creating core–sheath nanofibers [52,53,54,55], coaxial electrospraying is useful for generating core–shell micro- or nanoparticles [40,41]. A diagram shows the main components of a coaxial electrospraying apparatus, and the key elements in a spraying process are included in Figure 1. A concentric spraying head is the convergent place for the working fluids and the electrostatic energy. Two syringe pumps are exploited to quantitatively send the core and shell working fluids to the spraying head. A power supply is utilized to generate the applied high voltage, and a homemade collector is utilized to collect the deposited particles under the spraying head. Besides these four essential parts, often, a camera can be added to monitor the working process. During the working process, the first key is to form a Taylor cone, which is followed by the Coulomb explosion [40,41]. The second key is to achieve the solid particles, i.e., the designed products, after the effective removal of the solvents. A failed electrospraying process often results in a wet film. By the way, all the components of the electrospraying apparatus should be grounded for the safe operation of an electrospinning system [56,57].

In the traditional coaxial electrospraying process, the shell fluid should be solidable when it experiences an electrospraying process for ensuring the formation of solid core–shell particles. However, Li et al. reported that pure organic solvent (a non-solidable property) can be explored for creating high-quality nanoparticles [40]. Along this direction, the present work developed a modified coaxial electrospraying method, in which the dilute unsolidable SA solution was explored as the shell liquid to coat the core solidable EC-TC solution. The parameters for the electrospraying processes are included in Table 1.

**Figure 2:**
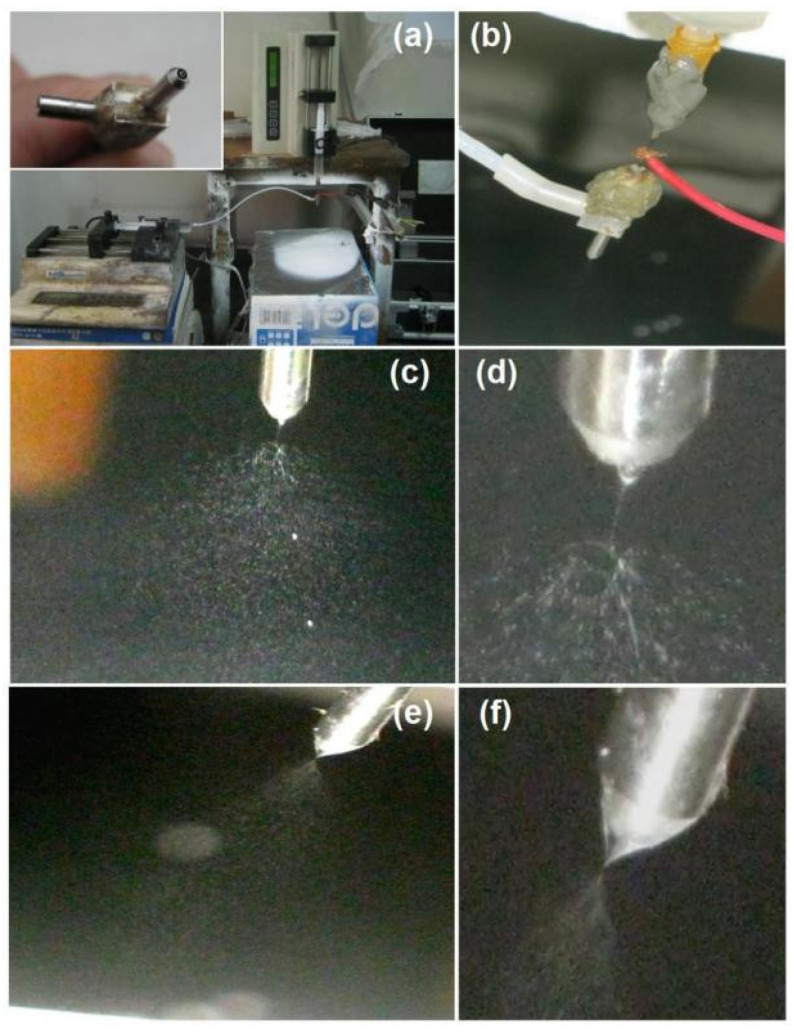
The coaxial electrospraying apparatus and observations of the working processes: (**a**) a digital picture of the electrospraying apparatus, with the upper left inset showing the concentric spraying head; (**b**) a digital picture showing the connection of the working fluids and the transferring of electrostatic energy; (**c**,**d**) a single-fluid electrospraying process experienced by the core solidable TC-EC solution under different magnifications for preparing the microparticles M1; (**e**,**f**) the digital photos of modified coaxial electrospraying processes for observing the whole process and the compound Taylor cone for producing the microparticles M2, respectively.

The key points about the implementation of electrospraying are recorded in Figure 2. A whole image of the modified coaxial electrospraying system is shown in Figure 2a. The homemade spraying head is exhibited in the upper left inset, which can also be explored in coaxial electrospinning and modified coaxial electrospinning. A coaxial outlet of the nozzle was utilized to guide the core and shell working fluids into the electrical field. The homemade collector was prepared by placing aluminium foil on a cardboard box. The working distance between the collector and the nozzle of the spraying head was fixed at 20 cm. The transportation of electrostatic energy and working fluids is given in Figure 2b. The copper line could be directly wrapped around the stainless steel capillary guiding the core fluid to transfer the high voltage. The core fluid was delivered to the spraying head directly by its syringe fixed on a pump. The shell fluid was sent to the inlet of the spraying head by the polymeric tubes, which were composed of a hard Teflon capillary and a section of highly elastic silicon tube as a connection between it and the metal inlet of the spraying head. This arrangement was exploited to avoid the absorbance of dichloromethane (DCM) and swelling of the silicon tube during the working process.

The applied voltage was adjusted to a suitable value based on a continuous and robust electrospraying process. When one of the two working fluids was switched off, then the coaxial electrospraying process downgraded into a single-fluid electrospraying process. As indicated by Figure 2c,d, for creating microparticles M1, a trio emerged between the Taylor cone, the straight fluid jet, and the Coulomb explosion, and the Taylor cone from the inner metal capillary was stable and in a typical cone shape. A high voltage of 21 kV is needed due to the higher surface tension of the core TC-EC fluid for a fluid rate of 1.0 mL/h.

After some optimization, the shell and core fluid flow rates were selected as 0.4 and 1.0 mL/h, respectively. The applied voltage was 18 kV. Although there was a generally larger fluid flow rate from the concentric nozzle (1.0 + 0.4 = 1.4 mL/h) for the modified coaxial process compared to the single-fluid electrospraying of the core fluid, the applied voltage was reduced to a smaller value of 18 kV compared to 21 kV. This strange phenomenon occurred because the Coulomb explosion is always initiated at the surface of the working liquid, and the electrostatic charges tend to gather on the surface of a working liquid. Thus, the smaller applied voltage was a direct result of the smaller surface tension of the shell SA solution. The working processes for generating microparticles M2 are recorded in Figure 2e (a typical whole coaxial electrospraying process) and Figure 2f (the compound core–shell Taylor cone). The single-fluid electrospraying of the shell SA solution (switching off the core fluid) resulted in only wet films due to a diluted SA concentration. The exploration of unsolidable SA solution as the shell fluid had the following advantages, besides providing a hydrophobic coating on the TC-EC medicated composites: (1) a good encapsulation of the core fluid at the nozzle of the spray head; (2) a facile initiation of the electrospraying process; (3) an easy fission of the sprayed droplets during the Coulomb explosion procedure; and (4) a stabler drying process of the core TC-EC solution, which would be demonstrated by the resultant products.

### 2.2. The Morphology and Structure of the Microparticles

**Figure 3:**
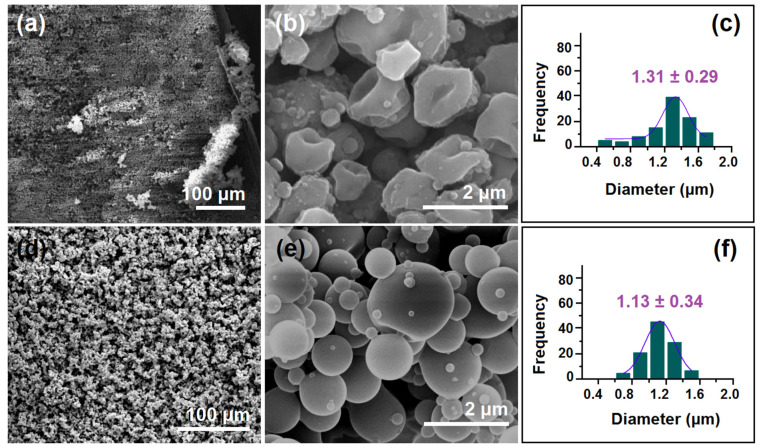
The morphologies and diameters of the electrosprayed microparticles: (**a**,**b**) SEM images of microparticles M1 at different magnifications; (**c**) the diameters of microparticles M1 and their size distributions; (**d**,**e**) SEM images of microparticles M2 at different magnifications; (**f**) the diameters of microparticles M2 and their size distributions.

The SEM images of the morphologies and the diameter distributions of microparticles from the single-fluid and modified coaxial processes are included in Figure 3. The differences between microparticles M1 and M2 can be concluded as follows: (1) particles M1 are dented from different directions and thus in an irregular shape (Figure 3a,b), whereas particles are mainly in a round shape (Figure 3d,e); (2) although both have satellites, particles M1 are more severe than M2; (3) particles M1 had a larger average diameter value (1.31 ± 0.29 μm, Figure 3c) than particles M2 (1.13 ± 0.34 μm, Figure 3f), although the applied voltage in producing M1 was bigger than that in producing M2.

**Figure 4:**
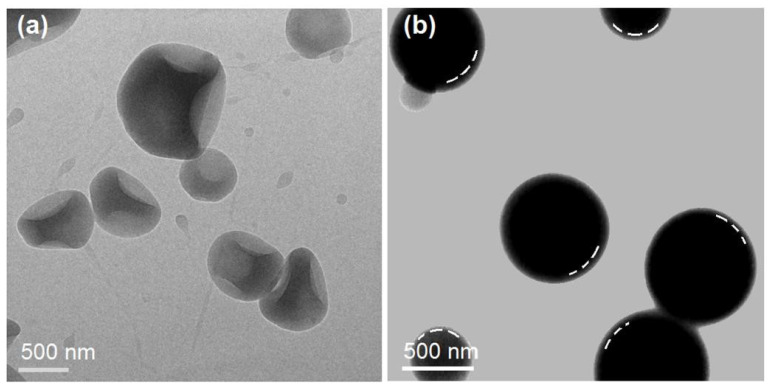
Inner structures of the electrosprayed microparticles: (**a**) TEM images of microparticles M1; and (**b**) TEM images of the microparticles M2.

The TEM images of particles M1 and M2 are included in Figure 4. In Figure 4a, it is obvious that microparticles M1 are irregular in their shape, with recessed sections at their surface. This indicates that the thicknesses of particles M1 varied, with no rules, which is reflected by the various gray levels in one particle, as the darker places mean a thicker region than the lighter gray places. In sharp contrast, microparticles M2 are mainly in a round shape, with a regular gray level change trend, i.e., the shell gray levels are always smaller than the core gray levels. By estimation, the thicknesses of these SA shell layers are between 10 and 30 nm.

**Figure 5:**
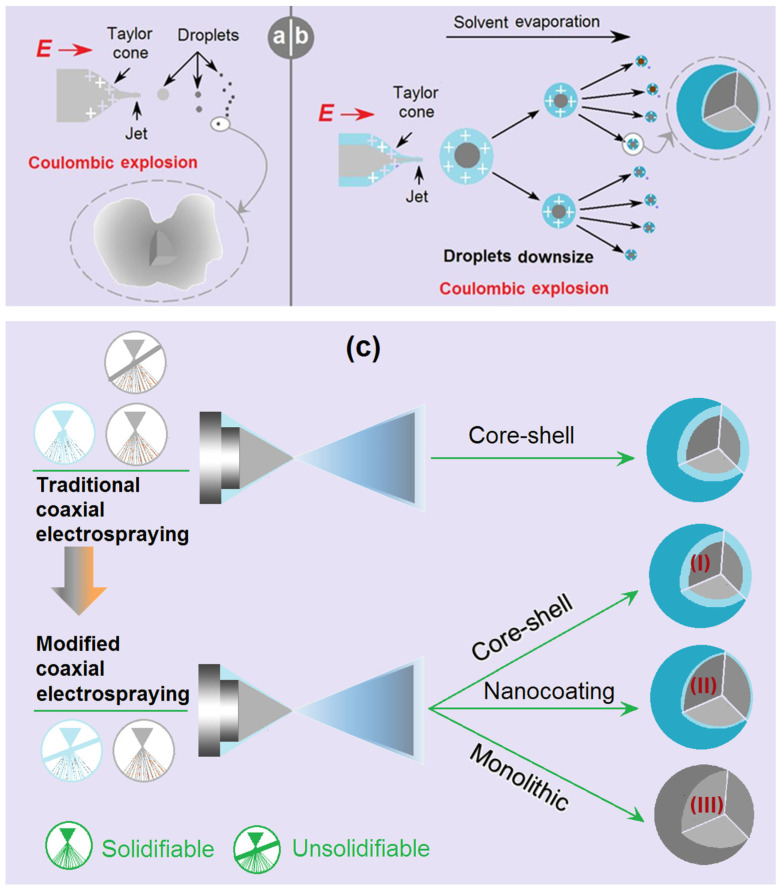
The microformation mechanisms: (**a**) the single-fluid electrospraying for producing microparticles M1; (**b**) the modified coaxial electrospraying for preparing microparticles M2; and (**c**) the different materials processing capabilities of traditional coaxial electrospraying and modified coaxial electrospraying processes; the case (II) represents the SA-coated core–shell microparticles M2 in this work.

A diagram showing the microformation mechanism of the single-fluid electrospraying process is exhibited in Figure 5a. The interaction between the electric energy, the surface tension, and the viscosity of the working fluid results in the Taylor cone, which is followed by a straight fluid jet and the Coulomb explosion. The most fundamental rule is that the “same charge repels each other,” by which the droplets are continuously splitting, reducing in volume, and being solidified to form the microparticles. During the process and at the late stage of the Coulomb explosion, the split droplets may have the semi-solid state of their surface but maintain a fluid state in the inner part. The formation of a solid film on the droplets not only retards further splitting but would also trap some solvent. Later, when the trapped solvent escapes to the environment, the barometric pressure would deform the round “droplet” shape to the irregular, dented shape in Figure 3b. This phenomenon has been reported in some other investigations about the preparations of electrosprayed CA particles [40] and zein nanoparticles [41].

When a diluted SA solution was exploited as a shell fluid to implement the modified coaxial electrospraying process, the shell solution, on the one hand, would dominate the Coulomb splitting process because of the surface distribution property of charges and its small surface tension (Figure 5b). On the other hand, the shell solvent would act as a bridge for the core solvents to move from the core sections to the atmosphere, and a more continuous and robust drying process could be ensured. Meanwhile, the shell solution may help to resist the outer disturbances for a stabler and more robust microparticles generation process. These positive factors should be attributed to the formation of the round shape of microparticles M2, and they would make the density of microparticles M2 larger than that of microparticles M1.

Compared with traditional coaxial electrospraying, the modified coaxial process has a stronger capability to create different kinds of particulate micro products. Shown in Figure 5c, the main difference in the implementation is that the shell fluid must be solidable in the traditional coaxial electrospraying, whereas the shell fluid in a modified coaxial process is unsolidable. From a standpoint of created products, traditional coaxial electrospraying can only create core–shell particles. In contrast, modified coaxial electrospraying can create core–shell particles (Case I), coat the particles’ surfaces in a continuous manner (Case II, i.e., the microparticles M2 in this work) or in a discontinuous way, and be exploited to create monolithic nanoparticles (Case III), such as by using only solvent as a shell fluid. This last case has been demonstrated by some previous investigations [40,41].

### 2.3. The Physical State and Compatibility

**Figure 6:**
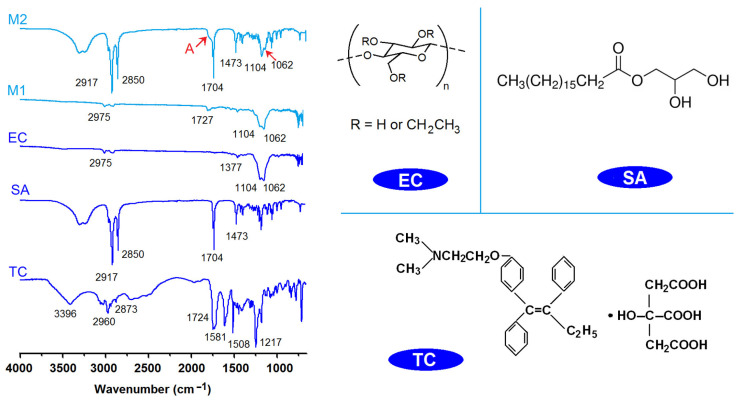
The ATR-FTIR spectra of the raw materials (TC, EC, and SA) and their electrosprayed microparticles (M1 and M2); the molecular formulas of EC, SA, and TC.

The ATR-FTIR spectra of the initial materials, i.e., EC, TC, and SA, and their electrosprayed microparticles M1 and M2, are included in the left section of Figure 6. The molecular formulas of EC, TC, and SA are given in the right side of Figure 6. TC spectra have a series of characteristic peaks, such as at 1724, 1581, and 1508 cm^−1^. SA has characteristic peaks at 1704, 2917, and 2850 cm^−1^, and EC’s characteristic peaks are at 1104 and 1062 cm^−1^. In the spectra of microparticles M1 from the single-fluid electrospraying process, the EC’s characteristic peaks are still clearly there. However, the characteristic peaks of TC are greatly reduced or even disappear, with only one peak distinguishable at 1727 cm^−1^. The reasons should be the formation of composites between EC and TC in the electrosprayed microparticles M1. In the spectra of microparticles M2, the peaks of SA are very obvious, which should be the reason that the detection depth of ATR-FTIR is about 10 nm. However, the information from the core section can still be discerned, such as the shoulder of the peak at 1704 cm^−1^ (as indicated by the “A” red arrow) and also the peaks at 1104 and 1062 cm^−1^. These results suggest that the shell SA and the core TC-EC composites co-existed in the core–shell particles in a hybrid manner, concurring with the TEM observations in Figure 4b. From the molecular formula of EC, SA, and TC, it can be anticipated that these components are highly compatible due to the secondary interactions between their molecules, such as hydrogen bonds, hydrophobic interactions, electrostatic interactions, and the van der Waals interaction [58,59].

**Figure 7:**
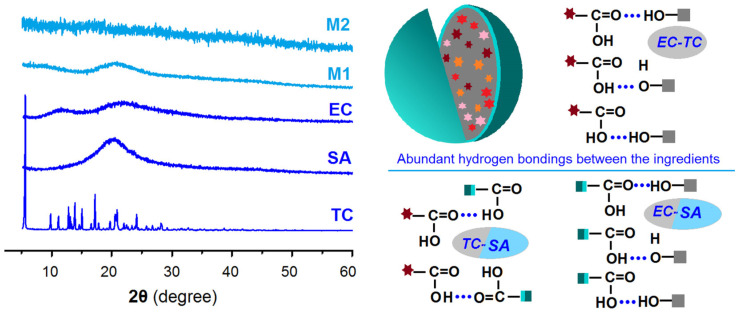
XRD patterns of the raw materials (TC, EC, and SA) and their electrosprayed microparticles (M1 and M2); the potential hydrogen bonds among the three components in the core–shell microparticles M2.

The XRD patterns of the raw materials of EC, TC, and SA, and their electrosprayed microparticles M1 and M2, are included in the left section of Figure 7. All the samples except the raw TC powders present in an amorphous state. TC patterns have some sharp Bragg peaks, suggesting their raw crystalline state. However, when TC was experiencing the electrospraying processes, regardless of whether the single-fluid monoaxial or the modified coaxial process was used, TC was converted into an amorphous state within its carrier EC in both monolithic composite microparticles M1 and core–shell hybrid microparticles M2. The electrospraying process is a very rapid fluid drying process, essentially. The short drying time period leaves almost no time for the TC molecules in the working fluids to recrystallize into new particles. The homogeneous state of the solutions was sufficiently maintained after the removal of organic solvents. Because there are abundant favorable secondary interactions between the components, the homogeneous state can be stably maintained. Shown in the right section of Figure 7 are the possible hydrogen bonds between EC and TC in the core section, between EC and SA, and between TC and SA in the core–shell interfaces.

### 2.4. The Drug Encapsulation Ratio of the Dual-Stage Drug Controlled-Release Profile

The measured entrapment efficiency (*EE*%) of TC was 99.4 ± 3.5% and 100.1 ± 3.3% for microparticles M1 and M2, respectively. The results indicate that all of the drug TC was successfully encapsulated into the microparticles through the electrospraying processes, regardless of whether the single-fluid process for creating particles M1 or the double-fluid modified coaxial process for generating the particles M2 was used. During the rapid electrospraying process, the TC solutions were converted into solid microparticles because of the evaporation of volatile solvents of ethanol and DCM to the environment. The solutes TC, EC, and also SA were solidified together, with few chances to escape from the working processes to the environment. The fate of a drug molecule from the preparation of its dosage form to its final clinical application is influenced by many factors [60,61,62,63,64]. Compared with many “bottom–up” nanofabrication methods, EHDA has its advantages to create drug-loaded micro/nano products with a higher *EE*% value.

**Figure 8:**
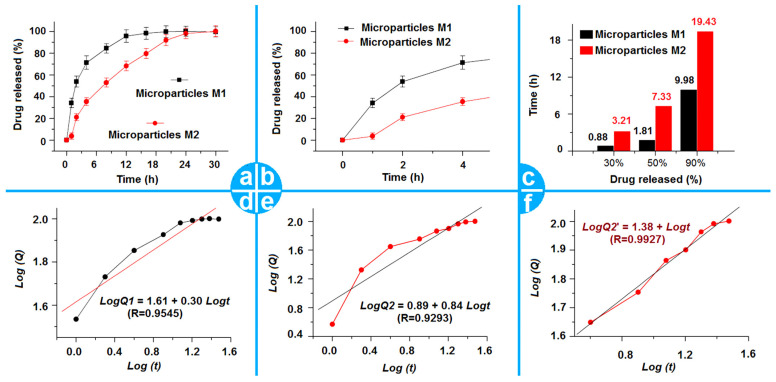
The drug sustained-release functional performances and the related drug controlled-release mechanisms: (**a**) The full time period drug sustained-release profiles of microparticles M1 and M2; (**b**) The first 4 h drug release profiles of the particles M1 and M2; (**c**) The regressed drug release time periods for releasing a certain percentage (30%, 50%, and 90%) of TC from the microparticles M1 and M2; (**d**) The regressed drug release mechanism for microparticles M1; (**e**,**f**) The regressed drug release mechanisms for microparticles M2 for the whole time period and from the 4th hour to the final time point, respectively.

The in vitro drug release profiles of microparticles M1 and M2 are exhibited in Figure 8a–c. Figure 8a shows the full time period experimental results of two kinds of microparticles. The comparisons between the two kinds of particles’ release profiles can help discern that the microparticles M2 from the modified coaxial process showed an obvious improved effect of the TC sustained release performances. In Figure 8a, it is clear that microparticles M2 showed a smaller tailing-off release phenomenon at the end of in vitro release, which is a negative phenomenon in drugs’ sustained-release profile. The initial burst release is another negative phenomenon in drug sustained release. Figure 2b shows that the initial burst release from the microparticles M1 is obvious. The microparticles M2 had no initial burst release. It is after almost 4 h that microparticles M2 released a drug release amount that matched what was released at the first hour from the microparticles M1. A further regressed treatment of the experimental data can calculate the time needed to release a certain percentage of drug from the microparticles. The results are included in Figure 8c. The release times for core–shell microparticles M2 for releasing 30%, 50%, and 90% of the loaded drugs were 3.21, 7.33, and 19.43 h, respectively. These results are better than those from the monolithic microparticles M1, whose corresponding values were 0.88, 1.81, and 93.98 h, respectively.

To further determine the drug release mechanisms from the two sorts of microparticles, the Peppas Equation (Q = kt^n^; Q, k, t, and n represent the accumulative drug release amount, a constant, the sampling time point, and the exponent [65]) was exploited to regress the in vitro drug release data. The results are shown in Figure 8d for microparticles M1 and in Figure 8e,f for microparticles M2. Just as expected, TC released from the microparticles M1 was manipulated by a typical Fickian diffusion mechanism. The regressed equation is LogQ1 = 1.61 + 0.30 Logt (R = 0.9545), in which the exponent value is 0.30, which is smaller than the judge standard of 0.45. Unexpectedly, the full time period regressions of microparticles M2 indicated a combination of diffusion and erosion mechanisms of TC release. The corresponding equation is LogQ2 = 0.89 + 0.84 Logt (R = 0.9293), which is shown in Figure 8e. The exponent is 0.84, between 0.45 and 0.90. However, when the data in the treatment are started from the fourth hour, a new regressed equation is achieved as LogQ2′ = 1.38 + 0.43 Logt (R = 0.9927). These results suggest that the shell SA had exerted a remarkable influence on the TC released from the core–shell microparticles M2, particularly during the first several hours. When the shell SA was removed at about 4 h, the drugs released from the core TC-EC composites were still manipulated by the typical Fickian diffusion mechanism, an anticipated result of drug molecules released from their insoluble matrices [66,67,68,69].

### 2.5. The Proposed Drug Controlled-Release Mechanism Based on the Insoluble Gel Forming

**Figure 9:**
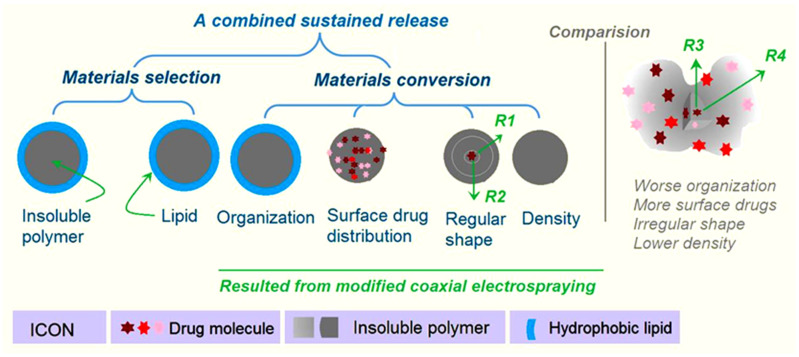
A sketch showing those factors that have co-acted to promote an improved sustained release of TC from the electrosprayed microparticles M2, in which a core insoluble gel composite is coated by a lipid shell layer.

Initially, polymer properties are the most important factor for providing a wide variety of drug controlled-release profiles, including drug sustained release [70,71,72,73,74]. Numerous reports in the literature have demonstrated that insoluble and biodegradable polymers and their composites and hybrids can be exploited as carriers to manipulate the gradual release of the loaded drug molecules [75,76,77,78]. Meanwhile, lipid materials are also frequently explored for the extended release of drugs in the formation of microparticles, micelles, composites, solid nanoparticles, emulsions, and liposome [79,80,81,82,83]. Thus, firstly, in this study, both insoluble polymer EC gels and the lipid SA were selected as the TC carrier, which are materials selected from experiences with the traditional pharmaceutics. Secondly, SA was exploited as a shell coating material to cover the polymer EC and the loaded drug molecules. This core–shell organization format at the micro scale is difficult to realize through the traditional chemical and physical methods. The coaxial electrospraying process proceeds in a one-step, straightforward manner. Meanwhile, modified coaxial electrospraying can organize the core–shell particles in a more regular, round shape. In contrast, the EC-TC particles M1 from the single-fluid electrospraying process have an irregular shape, which means an even more enlarged surface area for aggravating the initial burst release (Figure 9). Thirdly, the blank SA coating can make it so that there are no drug molecules on the surface of the electrosprayed microparticles, and thus completely eliminates the initial burst release phenomenon. Fourthly, the regular shape of microparticles M2 not only determines a relatively small surface for drug distribution and the amount of the drug initially released, but also determines a regular long diffusion distance for the penetration of both water molecules and drug molecules (e.g., routes R1 and R2 in Figure 9). In the irregular microparticles M1, the drug molecules would always diffuse to the dissolution bulk solution through the thinnest places (e.g., Route R3 is more possible than route R4), which is negative for the drug’s sustained release. Fifthly, although the fluid processing capacity per unit time in the modified coaxial electrospraying process is larger than that of the single-fluid electrospraying, the microparticles M2 are smaller than M1. This means that the particles M2 have a greater density than M1. The drug release route comprises water molecules penetrating into the EC-TC composites; absorbance of water, gelling, and swelling of EC molecules; dissolution of TC from the EC-TC composites; and diffusion of TC molecules from the inside of the particles to the bulk dissolution solutions. The greater density thus means a slower process of the above-mentioned procedure, which is favorable for the sustained release of TC. Thus, the fine sustained-release effect of microparticles is the result of multiple factors co-acting. One is the reasonable selection of the starting materials. The other four reasons come from the materials conversions through modified coaxial electrospraying. EHDA processes, as the popular material conversion method currently, hold many new approaches for creating novel, functional materials by updating the working strategies, such as the design of a biomimetic spraying head [84], exploring the alternating current [85,86], and integrating the chemical reaction into the physical drying procedure [87]. Meanwhile, the microformation mechanisms of these new EHDA processes deserve further investigations, which are completely different with those “bottom–up” fabrication processes, such as assemblies [88,89].

## 3. Conclusions

In this study, a modified coaxial electrospraying process was developed to encapsulate the anticancer drug TC in the core section of a new type of core–shell microparticle, in which the shell sections are the lipid SA coatings. Although the shell SA solution had no solidable property alone, it could ensure a robust and continuous modified coaxial electrospraying process to fabricate high-quality core–shell microparticles M2. SEM and TEM results demonstrated that M2 had a round shape, an obvious core–shell structure, and an estimated diameter of 1.13 ± 0.34 μm. XRD and FTIR verified that the drug TC presented in the electrosprayed products in an amorphous state, and TC had fine compatibility with EC and also SA. Compared with the electrosprayed TC-EC microparticles M1, the electrosprayed SA-coated microparticles M2 were able to provide an improved TC sustained-release profile. In total, 30% and 90% of the loaded drug sustained-release time periods were extended to 3.21 h and 19.43 h for M2, respectively, which was significantly longer than those provided by M1 (0.88 h and 9.98 h, respectively). Both the microformation mechanism of the modified coaxial electrospraying and the drug sustained-release mechanism from the core–shell microparticles M2 are suggested. The present study pioneered a brand-new manner for developing sustained drug delivery hybrids through a combination of insoluble cellulose gels and lipid using a modified coaxial electrospraying process.

## 4. Materials and Methods

### 4.1. Materials

TC with a purity greater than 99% was purchased from Shanghai Haosheng Bioengineering Company (Shanghai, China). The polymer EC and lipid SA were bought from Shanghai Huashi Big Pharmacy (Shanghai, China). The organic solvents DCM and anhydrous ethanol were analytical grade and obtained from Shanghai First Reagent Factory (Shanghai, China). Water was double distilled just before use.

### 4.2. Electrospraying

The homemade electrospraying apparatus comprised two syringe pumps (KDS100, USA) and a high voltage generator (ZGF2000/6 mA, Wuhan Huatian, Wuhan, China). The concentric spray head and the collector were homemade. The collecting distance from the nozzle of the spray head to the collector was fixed at 20 cm. The collected powders were kept in a desiccator until the characterizations.

### 4.3. Characterization

#### 4.3.1. Morphology and Inner Structure

A filed-emission scanning electron microscope (Quanter 450, FEI, Hillsboro, OR, USA) was used to evaluate the morphologies of microparticles M1 and M2. Before the assessments, the collected samples were placed on the conductive adhesives and sputtered with a thin layer of Pt. The applied voltage was fixed at 10 keV. The average diameters of the microparticles were evaluated using ImageJ software V1.8.0 (National Institutes of Health, Bethesda, MD, USA) by randomly measuring 100 places in the SEM images.

A transmission electron microscope (TEM, JEM2100F, JEOL, Tokyo, Japan) was used to evaluate the inner structures of microparticles M1 and M2. The samples were prepared by placing a carbon film supported by 200 × 200 Cu Mesh on the collector to collect microparticles for 1 min. The operational voltage was 300 keV.

#### 4.3.2. Physical State and Compatibility

A Spectrum 100 FTIR Spectrometer (Perkin-Elmer, Billerica, MA, USA) was used to conduct the ATR-FTIR detection. The samples included the raw TC, EC, and SA powders, and their electrosprayed products, microparticles M1 and M2. A Bruker X-ray Diffractometer (Karlsruhu, Germany) was utilized to achieve the XRD patterns of raw TC, EC, and SA powders and the electrosprayed microparticles M1 and M2. The X-rays were emitted at 40 kV and 30 mA. The recorded range of 2θ was between 5° and 60°.

### 4.4. Functional Performances

#### 4.4.1. Entrapment Efficiency

TC has a maximum absorbance at λ_max_ = 278 nm, which was exploited for its quantitative measurements. The *EE*% was measured by extracting the TC from the prepared microparticles M1 and M2 as follows: an accurately weighted product of microparticles was dissolved into a mixture of ethanol and DCM (6:4 in volume); then, 1 mL of the solution was dripped into 1000 mL of distilled water; after being centrifuged at 5000 rpm for 8 min at room temperature, the supernatant was measured using a UV-vis Spectrophotometer (UV-2102PC, Unico Instrument Co. Ltd., Shanghai, China). The encapsulated TC in the electrosprayed products could be calculated through the predetermined calibration standard equation. The value of *EE*% could be achieved through the following Equation (1):

(1) EE(%)=WmWp×100%

where *EE*% is the entrapment efficiency, *W_m_* is TC measured in the microparticles, and *W_p_* represents the TC added in the working fluids. All measurements were conducted in triplicate.

#### 4.4.2. In Vitro Dissolution Tests

The paddle method in the Chinese Pharmacopoeia (2020 Ed.) was explored to measure the in vitro release profiles of microparticles M1 and M2. The phosphate buffer solution (PBS, 0.01 M, pH 7.0) was utilized as the dissolution bulk media. The experimental conditions included 600 mL of PBS, 37 °C, and a rotation rate of 50 rpm. At predetermined time points, a 5.0 mL aliquot was withdrawn, and 5.0 mL of fresh PBS solution was added. All in vitro experiments were repeated six times.

## Figures and Tables

**Table 1:** Fabrication parameters of the microparticles.

No.	Electrospraying	Applied Voltage (kV)	Fluid Flow Rate (mL/h)		Products
			Core ^a^	Shell ^b^	
M1	Single fluid	21	--	1.0	Monolithic microparticles
M2	Coaxial	18	0.4	1.0	Core–shell microparticles
M3	Single fluid	12	0.4	--	--

^a^ The core fluid consisted of 2.0 g of TC and 10.0 g of EC in 100 mL of solvent mixture of anhydrous ethanol and DCM with a volume ratio of 6:4. ^b^ The shell solution was composed of 1.0 g of stearic acid in 50 mL of DCM.

## Data Availability

The data supporting the findings of this manuscript are available from the corresponding authors upon reasonable request.
